# TritiKBdb: A Functional Annotation Resource for Deciphering the Complete Interaction Networks in Wheat-Karnal Bunt Pathosystem

**DOI:** 10.3390/ijms23137455

**Published:** 2022-07-05

**Authors:** Naveen Duhan, Raghav Kataria, Rakesh Kaundal

**Affiliations:** 1 Department of Plants, Soils, and Climate, College of Agriculture and Applied Sciences, Utah State University, Logan, UT 84322, USA; naveen.duhan@usu.edu (N.D.); raghav.kataria@usu.edu (R.K.); 2 Bioinformatics Facility, Center for Integrated BioSystems, College of Agriculture and Applied Sciences, Utah State University, Logan, UT 84322, USA; 3 Department of Computer Science, College of Science, Utah State University, Logan, UT 84322, USA

**Keywords:** effector proteins, domain, interolog, Karnal bunt, wheat, protein-protein interactions, *Tilletia*, transcription factors, *Triticum*

## Abstract

The study of molecular interactions, especially the inter-species protein-protein interactions, is crucial for understanding the disease infection mechanism in plants. These interactions play an important role in disease infection and host immune responses against pathogen attack. Among various critical fungal diseases, the incidences of Karnal bunt (*Tilletia indica*) around the world have hindered the export of the crops such as wheat from infected regions, thus causing substantial economic losses. Due to sparse information on *T. indica*, limited insight is available with regard to gaining in-depth knowledge of the interaction mechanisms between the host and pathogen proteins during the disease infection process. Here, we report the development of a comprehensive database and webserver, *TritiKBdb*, that implements various tools to study the protein-protein interactions in the *Triticum species*-*Tilletia indica* pathosystem. The novel ‘interactomics’ tool allows the user to visualize/compare the networks of the predicted interactions in an enriched manner. *TritiKBdb* is a user-friendly database that provides functional annotations such as subcellular localization, available domains, KEGG pathways, and GO terms of the host and pathogen proteins. Additionally, the information about the host and pathogen proteins that serve as transcription factors and effectors, respectively, is also made available. We believe that *TritiKBdb* will serve as a beneficial resource for the research community, and aid the community in better understanding the infection mechanisms of Karnal bunt and its interactions with wheat. The database is freely available for public use at http://bioinfo.usu.edu/tritikbdb/.

## 1. Introduction

Wheat (*Triticum* spp.) is the most important staple crop of the world, and a significant source of various nutrients, including proteins, vitamins, carbohydrates, lipids, and other minor nutrients, and host to a wide range of fungal pathogens [1,2]. Recently, infectious diseases and pests have become a considerable concern in wheat production, owing to about 21.5% of yield loss, depending on environmental conditions and the pathogen’s virulence. Fungal pathogens belonging to *Puccinia*, *Ustilago*, and *Tilletia* species are considered the primary cause of biotic stress in wheat. They are responsible for considerable losses in the quality and quantity of the crop [3]. The quarantine disease in wheat, Karnal bunt (KB), is caused by *Tilletia indica* and has emerged in various countries worldwide, including India, Mexico, South Africa, and the USA [4]. The disease is primarily seed-borne, but the teliospores can also spread through soil or air, thus affecting the grain quality. Following previous reports, the annual yield loss of the disease ranges from 0.5% to 1%. Still, KB is recognized as having a severe economic impact due to the loss of grain weight (about 0.25%), followed by the imposition of various international regulatory/quarantine restrictions on the crop grown in KB-infected regions [5]. Most countries have a zero tolerance for Karnal bunt in import shipments.

The morphological and physiological variability in *T. indica* isolates enhance their ability to infect a wide range of hosts; thus, they are considered genetically variable for developing resistant crop varieties [6]. Several methods, such as disease diagnosis in the field, immunodiagnostic assays, and molecular techniques, have been implemented to detect the disease, but these have different constraints. For example, disease detection in the field is challenging due to the production of a low number of bunted kernels in each spike. At the same time, the molecular techniques are expensive and time-consuming [7,8,9].

Molecular interactions form the basis of pathogenicity. The protein-protein interactions (PPIs) between host and pathogen are highly dynamic throughout the disease infection mechanism [10,11,12]. The pathogens secrete effector proteins for successful invasion of host cellular defense mechanisms. In contrast, the host activates a suite of immune responses such as PAMP-triggered immunity (PTI), effector-triggered immunity (ETI), hypersensitive response (HR), and other metabolic pathways against the pathogen attack [13,14]. Studying these interactions between host and pathogen proteins is vital to researchers. Also, due to the limited information on pathogen pathogenicity-related biomarkers, no successful *Triticum* cultivar resistant to KB has been developed [15]. We developed a database, *TritiKBdb*, using two computational approaches, interolog-based and domain-based, to predict the protein-protein interactions between two widely grown wheat species (*T. aestivum* and *T. turgidum*), and *T. indica*. This database is the first open-source platform which allows the research community to predict protein-protein interactions and obtain the functional annotations of the host and pathogen proteins to better understand the disease infection mechanism of Karnal bunt in *Triticum* species. *TritiKBdb* aims to provide a platform for comparative study of the *Triticum* species proteins on infection with Karnal bunt. One could compare and visualize these interaction networks in an efficient and enriched visualization environment. *TritiKBdb* is freely accessible at http://bioinfo.usu.edu/tritikbdb/.

## 2. Results and Discussion

### 2.1. The Compendium

In the past decade, there has been a substantial increase in infectious diseases in crops, leading to reduced yield and crop quality. Understanding the infection mechanism requires in-depth knowledge of the functioning of the host and pathogen proteins and the cellular environment where the interaction between the host and pathogen proteins occurs. The molecular techniques to study these interactions are efficient but time-consuming and expensive. Integrating computational approaches with comprehensive systems biology can enhance the study of protein-protein interactions [16,17,18]. The primary goal of developing *TritiKBdb* is to provide the research community with a comprehensive platform containing the respective functional annotations of the host and pathogen involved in the protein-protein interactions during the Karnal bunt disease infection in wheat. The database implements different tools to predict the inter- and intra-species PPIs between *Triticum* species and *Tilletia indica* and further study the interactions using the protein annotations available in various database modules.

### 2.2. Interactome Prediction Tool

**Figure 1:**
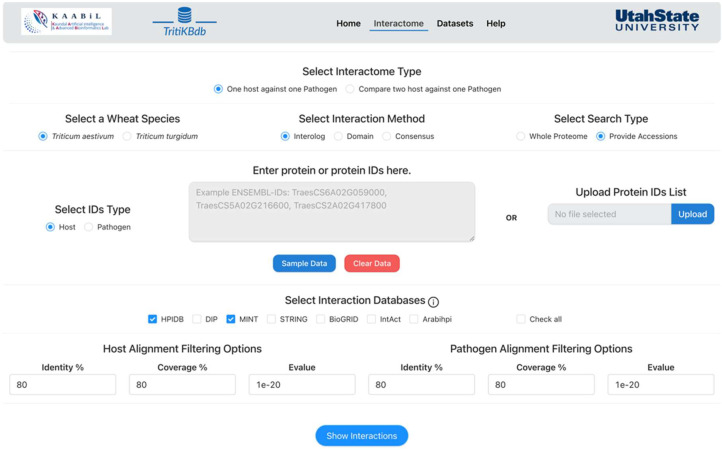
The Interactome search and Comparison tool of *TritiKBdb*.

**Figure 2:**
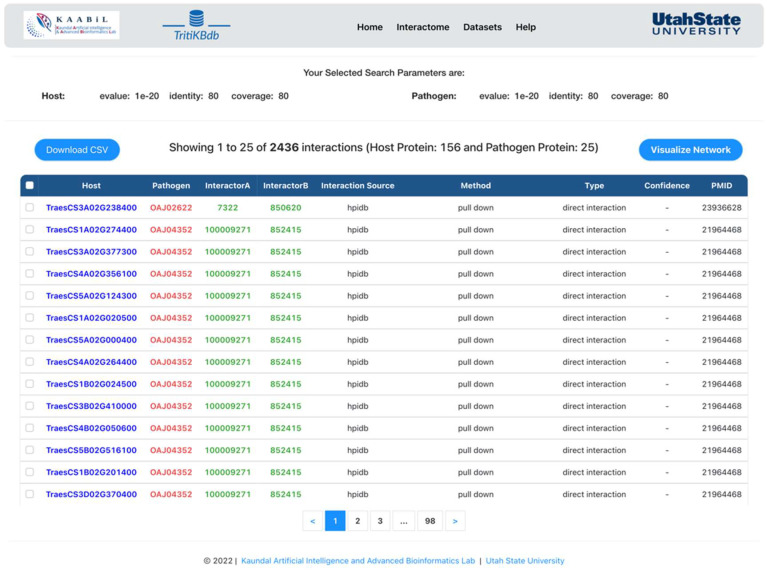
The Interaction Results page of *TritiKBdb*.

We implemented a comprehensive ‘Interactomics’ tool to predict the experimentally validated protein-protein interactions between *Triticum* species and *T. indica* (Figure 1). This tool allows the user to predict the host-pathogen interactions based on various gold standard databases (mentioned in Materials and Methods). The user can select any interaction category (Interolog/Domain/Consensus), choose the available interaction databases, and specify the alignment filtering values such as sequence identity (%), sequence coverage (%), and e-value to obtain the interactions. The resulting interactions include the host-pathogen interaction pair from the source interaction databases, experimental method used for interaction validation, interaction type (physical or direct), confidence score, and PMID of the publication for the validated interactions linked to PubMed (Figure 2). All the proteins are linked to their respective external resources (Ensembl, UniProt, NCBI, etc.) to provide more information to the user.

**Figure 3:**
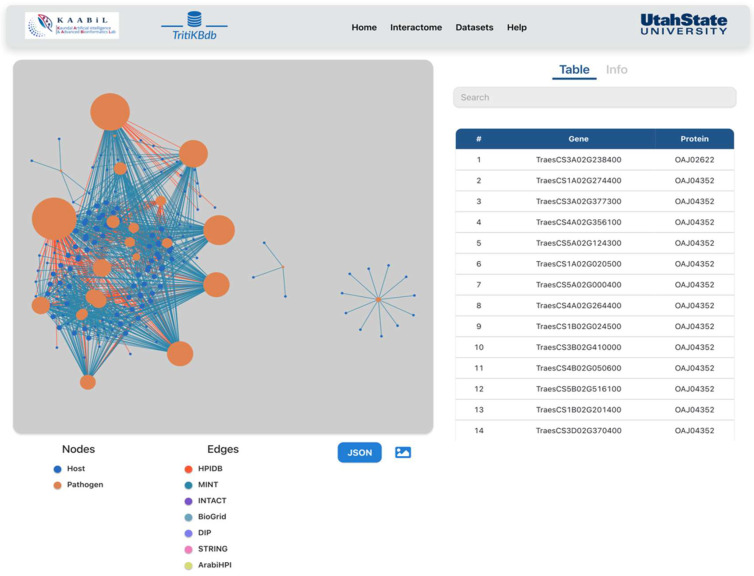
Network of resulting interactions from the host-pathogen interactome module. Node size represents the degree.

Users can visualize the resulting interactions with the network module, where the size of the nodes represents the degree of the protein. The user can also visualize the network of the interactions of interest. The user can view a specific interaction in the network by selecting a distinct edge between two proteins. The user can also download the network in ‘JSON’ format for advanced visualization, which is the go-to format for various network visualization tools. *TritiKBdb* provides the user a distinct functionality to predict the interactions in two ways: (i) PPI prediction for one host against one pathogen and (ii) comparison of PPIs for two hosts against one pathogen. In the ‘one host against one pathogen’ option, the user can select a host (*T. aestivum* or *T. turgidum*) and pathogen (*T. indica*) to predict the interactions using the available parameters, while in the ‘two hosts against one pathogen’ option, the user can obtain the interactions in two ways. The first method is through unique interactions with only one host. These interactions are host-specific and include the *Triticum* proteins that are not the orthologs of each other. The second method is used when the interactions are common to both hosts. These interactions are predicted based on the *Triticum* species that are orthologs of each other. This functionality of *TritiKBdb* enables the user to study the species-specific interactions and the interactions that are common from both the host species, thus making it highly efficient for the users to compare two *Triticum* species simultaneously (Figure 3).

### 2.3. BLAST Search

To enable the users to query the protein of interest for similarity against the *Triticum* species and *T. indica* proteomes, we implemented the BLAST (Basic Local Alignment Search Tool) search module on *TritiKBdb*. BLAST module uses in-house Python and JavaScript to perform the alignment. Users can visualize BLAST module results against selected proteomes and export the resulting alignment in a tab-delimited file.

### 2.4. Advanced Search

**Figure 4:**
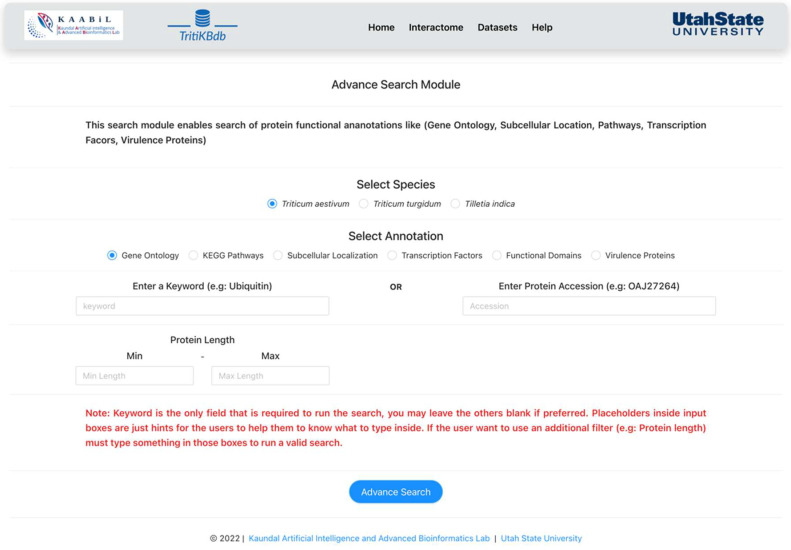
A snapshot of the ‘Advanced search’ module of *TritiKBdb*.

The advanced search module is a comprehensive module in *TritiKBdb* that allows the user to search the host and pathogen proteins based on specific keywords (Figure 4). These keywords include protein annotation terms, biological terms, protein length, subcellular localization of the protein, and genomic parameters (gene coordinates). The user can provide a keyword (mandatory) and other optional parameters, following which the tool will filter and provide the related proteins for the specific species selected. The tool will search for the provided keyword and other parameters in that same annotation with the chosen annotation.

This tool also provides the InterPro accessions associated with the proteins and an external link to the InterProScan website. This module is interconnected with other functional annotations of the proteins available on the database, thus making it extensive and time-efficient.

### 2.5. Functional Annotations

Functional annotation involves integrating the biological information into the proteins, which is crucial to understanding the molecular mechanisms of the disease infection. In addition to the host-pathogen PPI prediction, we aimed to provide the user with various functional annotations of the host and pathogen proteins involved in the predicted interactions. These annotations include protein subcellular localization, KEGG (Kyoto Encyclopedia of Genes and Genomes) pathways, available domains, gene ontology, host transcription factors, etc. These annotations were generated with the different tools mentioned above in the ‘Materials & Methods’ section.

The ‘functional annotation’ feature for all the species is available on the homepage of *TritiKBdb*. Some of the functional annotations, including subcellular localization, functional domains, KEGG pathways, and gene ontology terms, are available for both the host and pathogen proteins. The determination of the subcellular localization of the protein helps characterize its function in different cellular environments [19]. Since the experimental approaches are time-consuming [20], we implemented diverse computational tools to predict the subcellular localization of the host and pathogen proteins and provide them to users via the web interface. Being structurally and functionally conserved, the domains help the proteins perform various cell functions [21]. The functional domains for the host and pathogen proteins available on *TritiKBdb* represent the specific domain family and the description associated with the domain, along with the source domain database (Pfam, SMART, ProSiteProfiles, PANTHER, PRINTS, Gene3D, TIGRFAM, CDD, Superfamily). The KEGG pathways and Gene Ontology terms (along with their respective description) for the host and pathogen proteins are available on the database.

Further, the database also provides organism-specific annotations of the proteins. The proteins that serve as transcription factors (TFs) are available for *T. aestivum* and *T. turgidum*. Being the primary defense factors in plants, TFs play a crucial role in biotic and abiotic stresses by activating various defense-related molecular responses against pathogen attack [22]. The information about the transcription factor family of the proteins is also made available to the user. TFs available on *TritiKBdb* are 5833 and 3461 for *T. aestivum* and *T. turgidum*, respectively. As both of the *Triticum* species are highly similar concerning their genomes, we implemented the orthologs of *T. aestivum* and *T. turgidum*. We implemented the modules that contain the information about the pathogen proteins that serve as effector and secretory proteins for the pathogen proteins. A new feature module is also implemented whereby the pathogen proteins that serve as both effector and secretory proteins are available. These proteins subvert the host cell’s immune responses during the attack. Apart from this, the effector and secretory proteins also play a role in self-defense, obtaining nutrients from the host, and helping in niche colonization [23]. We believe that the functional annotations of the host and pathogen proteins provided in different modules will enhance the understanding of the disease infection.

### 2.6. Validation and Use Cases of TritiKBdb

We further validated the functional protein annotations in the *TritiKBdb* by integrating it with the disease resistance QTLs/SNPs reported in the literature. Researchers have identified numeral QTLs and SNPs on different chromosomes (1DL, 2BL, 2DL, 3BS, 4AL, 4B, 4D, 5AL, 5AS, 5BL, 6BL, 6BS, 7BS, 7DL) of wheat using genome-wide association studies (GWAS) [24,25,26,27]. We found 47,583 wheat proteins in the predicted interactions associated with the bunt-resistance-related chromosomes (Supplementary Material File S1). For database validation, a random host-pathogen interaction pair “TraesCS2D02G583100.1-OAJ04754” was selected from the predicted PPIs, and the proteins were annotated using the functional annotations available on *TritiKBdb*. The host protein in this pair was significantly involved in plant defense-related mechanisms such as biosynthesis of secondary metabolites and response to oxidative stress (GO:0006979) and belonged to the peroxidase domain family. In the previous studies, these mechanisms have been shown to play a crucial role in plant immune responses during biotic stress [28,29]. This protein’s subcellular localization was predicted as ‘plastid,’ which is a major site for the production of defense-related signals and hormones such as ABA, reactive oxygen species (ROS), and others [30]. In comparison, the interacting pathogen protein in the selected PPI pair was involved in betalain biosynthesis and the small molecule metabolic process (GO:0044281) responsible for fungal pathogenicity and development [31]. In correspondence with the literature, these annotations confirm the viability of the data and makes it a good candidate pair for further experimental validation.

#### HPIs of Experimentally Validated Karnal Bunt Virulence Proteins

**Figure 5:**
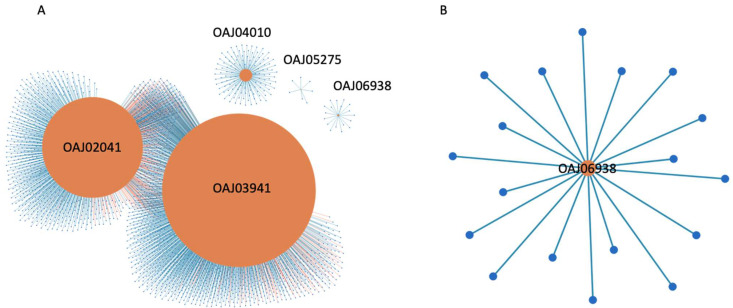
(**A**) Network visualization of 5 karnal bunt virulence proteins interacting with *T. aestivum* proteins. Node size (orange) is based on the degree of interactions, (**B**) Individual network visualization; shown here OAJ06938 interacting with 18 common wheat (*T. aestivum*) proteins.

To further validate the applicability of *TritiKBdb*, we selected some experimentally proven Karnal bunt virulence proteins from the literature [32] and searched those in our database to find their corresponding wheat interacting partners (receptors). About 10 *T. indica* experimentally identified virulence/pathogenicity factors were selected (OAJ02041, OAJ06104, OAJ04365, OAJ03869, OAJ04747, OAJ03941, OAJ05275, OAJ06938, OAJ04010, OAJ06388). The corresponding interactions of these proteins were identified using the ‘Search Interactome’ module on *TritiKBdb* in two databases (HPIDB and MINT; *users can select as many databases as they want*). We found five *T. indica* proteins (at identity > 30, coverage > 40 and e-value < 1 × 10^−4^) interacting with 2101 *T. aestivum* proteins resulting in 2548 unique HPIs (Supplementary Material File S2). The network of these resulting HPIs is shown in Figure 5A. Among these interactions, OAJ03941, OAJ02041, OAJ04010, OAJ06938, and OAJ05275 interact with 1379, 1022, 122, 18, and 7 *T. aestivum* proteins, respectively. These types of analysis with *TritiKBdb* can be extremely useful to experimental biologists, plant pathologists, and others when they are inferring functional HPIs within a system which can be directly implemented to develop disease resistant cultivars. Further, one could visualize the individual protein interaction networks on a zoomed scale; for example, OAJ06938 is interacting with 18 *T. aestivum* proteins and the resulting network for this protein is depicted in Figure 5B. The detailed information about the individual interacting pairs, their corresponding NCBI or Uniprot IDs, experimental evidence, confidence score, etc. information can be found in Supplementary Material File S2.

Similarly, users can also search the interactions against the durum wheat (*T. turgidum*) and compare the HPIs of Karnal bunt proteins with common wheat as well as durum wheat. Thus, *TritiKBdb* can play a significant role in identifying the interacting partners of *T. indica* virulence proteins with the corresponding *T. aestivum* and *T. turgidum* receptor proteins.

## 3. Materials and Methods

### 3.1. TritiKBdb Interface

**Figure 6:**
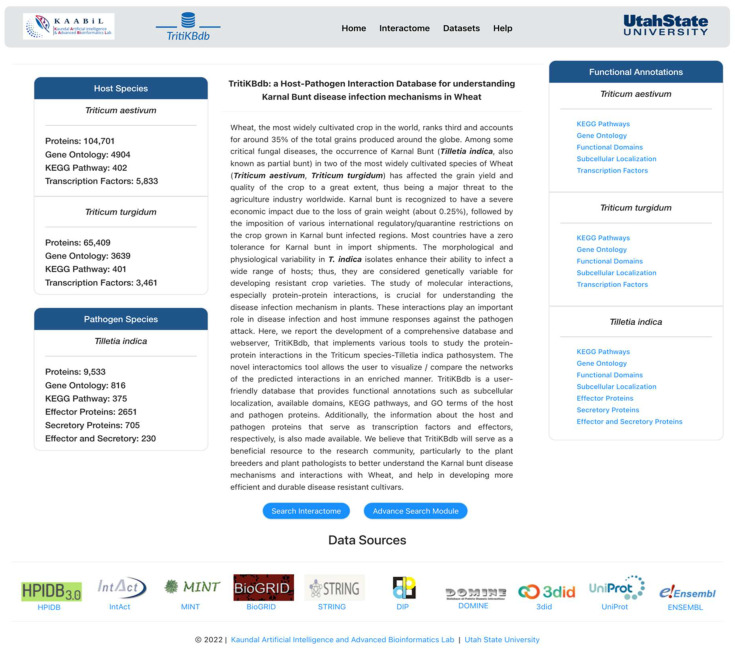
The ‘Home’ page of *TritiKBdb* database.

We developed a user-friendly database, *TritiKBdb*, which is freely available at http://bioinfo.usu.edu/tritikbdb/ (accessed on 9 June 2022) (Figure 6). The database was created using the MERN stack technology and served via NodeJS. MongoDB, a non-structured query language database, was used to store the interaction files, blast files, and functional annotations. The backend APIs were developed using ExpressJS v4.17 and NodeJS v16, and the frontend was developed using REACT. The “Help page” has also been made available for efficient browsing of the database for novice users. Additionally, an information icon on the tools/features page gives a piece of brief information about the functions and datasets available.

### 3.2. Dataset Collection and Processing

For the protein-protein interaction prediction, the proteomes of *Triticum* species (common wheat and durum wheat) were obtained from Ensembl Plants, while those of *Tilletia indica* were downloaded from Ensembl Fungi (Table 1). The complete proteome of each species was analyzed with CD-HIT (Cluster Database at High Identity with Tolerance) [33] at 100% identity to remove the identical sequences. In the case of *Triticum* species, the longest sequence of the proteins was considered, followed by analysis with CD-HIT. The information on the datasets used is available on the “Datasets” page of *TritiKBdb*.

Various functional annotations such as subcellular localization, effector proteins, secretory proteins, transcription factors, and functional domains were obtained using different tools (Table 2). Plant-mSubP and DeepLoc employ machine learning-based algorithms to predict the subcellular localization of proteins. EffectorP and SignalP were used to analyze whether a particular pathogen protein serves as an effector and secretory protein. To identify the proteins of *Triticum* species that are transcription factors, we employed PlantTFDB, which determines the transcription factor binding motifs in the promoter region. We also used InterProScan to obtain the functional domains of the host and pathogen proteins.

### 3.3. Working of the Interactomics Tool

The interolog and domain-based PPI predictions between *Triticum* species and *T. indica* were accomplished by implementing the gold standard databases (Table 3). These databases include several protein-protein interaction databases: BioGRID [40], DIP [41], HPIDB [42], IntAct [43], MINT [44], STRING [45], and domain-domain interaction databases: 3did [46], DOMINE [47], and IDDI [48]. We filtered these interaction databases for the experimental interactions based on the evidence such as literature support, interaction type/experimental method, and confidence score. For interolog-based prediction, the proteomes were analyzed with BLAST and searched against the PPI databases to obtain the alignments. At the same time, HMMER was used to extract the domains of the proteins of all the species for the domain-based approach. The resulting alignments and domains were then subjected to our in-house python scripts to predict the host-pathogen protein-protein interactions. This tool also provides the user to visualize the networks of the predicted interactions, for which we implemented Cytoscape [49]. The users can also download the predicted interactions in a tab-delimited format.

## 4. Conclusions

The host-pathogen protein-protein interaction database, *TritiKBdb*, was developed to provide the research community with a platform that helps gain in-depth knowledge about the Karnal bunt infection mechanisms in *Triticum* species. The database is built using an extensive framework and hosts various functional annotations of the proteins involved in the host-pathogen interactions. We implemented a customizable ‘interactomics’ tool, which predicts the PPIs based on user-provided parameters. The network visualization of the predicted interactome is displayed in an information-rich and interactive visualization pattern, and the layouts can be customized further based on user-defined schema. Furthermore, the extensive tool ‘advanced search’ is a hub of functional annotation information for the users. We believe *TritiKBdb* will serve as a potential resource to the scientific community, plant breeders, and plant pathologists, to aid them in better understanding the Karnal bunt disease using the protein-protein interaction information with wheat, and aid in the development of more efficient and disease resistant cultivars. In the future, we plan to add more species of wheat (and its pathogens) to the database as their whole genome sequencing datasets become available. Karnal bunt also infects Triticale, a hybrid of wheat and rye, but the whole genome sequences are not available yet for Triticale; when available, they could be added to the *TritiKBdb* resource too.

## Figures and Tables

**Table 1:** Dataset sources of host and pathogen proteomes.

Species	Source	Number of Proteins	
		Downloaded	CD-HIT
*Triticum aestivum*	Ensembl Plants (https://plants.ensembl.org/index.html, accessed on 8 April 2022)	133,346	104,701
*Triticum turgidum*	Ensembl Plants (https://plants.ensembl.org/index.html, accessed on 8 April 2022)	196,105	65,409
*Tilletia indica*	Ensembl Fungi (https://fungi.ensembl.org/index.html, accessed on 8 April 2022)	9548	9533

**Table 2:** Tools employed to obtain functional annotation of the host and pathogen proteins.

Functional Annotation	Tool	Link	Reference
Subcellular localization	Plant-mSubP (Host)	http://bioinfo.usu.edu/Plant-mSubP/ (Accessed on 14 April 2022)	[34]
	DeepLoc 1.0 (Pathogen)	https://services.healthtech.dtu.dk/service.php?DeepLoc-1.0 (Accessed on 15 April 2022)	[35]
Effector proteins	EffectorP 3.0	https://effectorp.csiro.au/ (Accessed on 11 April 2022)	[36]
Secretory proteins	SignalP 6.0	https://services.healthtech.dtu.dk/service.php?SignalP (Accessed on 11 April 2022)	[37]
Transcription factors	PlantTFDB v5.0	http://planttfdb.gao-lab.org/ (Accessed on 11 April 2022)	[38]
Functional domains	InterProScan	https://www.ebi.ac.uk/interpro/ (Accessed on 15 April 2022)	[39]

**Table 3:** Information of the databases employed inside *TritiKBdb*.

Database	Total Interactions	Link to Database
BioGRID	2,381,484	https://thebiogrid.org/ (accessed on 27 April 2022)
DIP	76,882	http://dip.doe-mbi.ucla.edu/ (accessed on 27 April 2022)
HPIDB	69,365	https://hpidb.igbb.msstate.edu/ (accessed on 27 April 2022)
IntAct	1,184,057	http://www.ebi.ac.uk/intact/ (accessed on 27 April 2022)
MINT	132,249	http://mint.bio.uniroma2.it/ (accessed on 27 April 2022)
ArabiHPI	983	Manually curated
STRING	4,313,229	https://string-db.org/ (accessed on 27 April 2022)
3did	11,200	https://3did.irbbarcelona.org/ (accessed on 27 April 2022)
DOMINE	26,219	https://manticore.niehs.nih.gov/cgi-bin/Domine (accessed on 27 April 2022)
IDDI	204,716	http://pcode.kaist.ac.kr/iddi/ (accessed on 27 April 2022)

## Data Availability

Not applicable.

## Supplementary Materials

The following supporting information can be downloaded at: https://www.mdpi.com/article/10.3390/ijms23137455/s1.
